# Deep learning optimization of STAR-RIS for enhanced data rate and energy efficiency in 6G wireless networks

**DOI:** 10.1038/s41598-025-09774-6

**Published:** 2025-07-20

**Authors:** Amal Megahed, Ahmed. M. Abd El-Haleem, Mahmoud M. Elmesalawy, Ibrahim I. Ibrahim

**Affiliations:** Department of Electronics and Communications Engineering, Faculty of Engineering, Helwan University, Cairo, 11792 Egypt

**Keywords:** Simultaneously transmitting and reflecting reconfigurable intelligent surfaces, Multi-input single-output, Deep learning, Spectral energy efficiency, Satisfaction rate, Spatial temporal analysis network, Engineering, Mathematics and computing

## Abstract

Unlike traditional reflection-only reconfigurable intelligent surfaces (RISs), simultaneously transmitting and reflecting RISs (STAR-RISs) introduce an innovative technology. They expand the coverage area from half-space to full-space. This advancement provides new degrees of freedom (DoF) for controlling and optimizing signal propagation. This research explores the performance of the STAR-RIS in 6G wireless networks, comparing nearly passive STAR-RIS (NP-STAR) and active STAR (ASTAR) against nearly passive RIS (NP-RIS) and active RIS (ARIS) benchmarks. Through extensive simulations and deep learning (DL)-based optimization, we assess achievable data rates and spectral energy efficiency (SEE) across various system configurations, training dataset sizes, and user locations. Results show that in high-interference environments, NP-STAR configurations can sometimes exceed ASTAR implementations due to their ability to mitigate interference amplification. In addition, the deterioration in spectral energy efficiency (SEE) of active implementations compared to nearly passive ones confirms their greater consumption of energy. The exploration of STAR-RIS technology in 6G networks reveals key research gaps in prior works. These include limited studies on performance under real-world conditions, insufficient understanding of energy-spectral efficiency trade-offs, and the need for reliable DL optimization across scenarios. Practical deployment issues like power management and interference control also lack adequate research. Addressing these gaps is crucial for advancing STAR-RIS technology in this paper. This study emphasizes the importance of managing transmit power levels at base stations to control interference, with active setups particularly excelling in optimal channel conditions. Furthermore, DL approaches can effectively approximate genie-aided performance bounds with adequate training data, especially in complex channel scenarios. These insights provide practical guidance for deploying STAR-RIS technology in demanding wireless networks.

## Introduction

Reconfigurable intelligent surfaces (RISs) have gained prominence as an innovative solution for improving wireless network coverage and boosting data rates^1–3^. Comprised of a planar array of cost-efficient passive elements, the RIS manipulates the phase and amplitude of signals through a smart controller, effectively redirecting them toward their intended targets. These antennas can be pre-programmed with a predefined redirecting codebook, allowing them to reflect signals along various paths. However, large-scale RIS deployments encounter significant challenges: accurate channel estimation becomes exceedingly complex due to the vast number of passive elements, while codebook design demands extensive channel state information (CSI), which is expensive to acquire^4^. Traditional methods often assume perfect CSI availability, focusing exclusively on beamforming matrix optimization while overlooking critical factors like spectral and energy efficiency. Deep learning (DL) presents a promising solution to overcome these challenges^5^. The design of a codebook for the beamforming matrix in RIS-aided communication systems is thoroughly explored in^6^ and related references, presenting various DL strategies. One method involves directly mapping channel conditions to optimal beamforming matrices. For instance, a deep neural network (DNN) architecture with two stages is employed in^7^, where the first DNN estimates channel state information (CSI), and the second predicts optimal phase shifts and beamforming vectors. These DNNs typically consist of fully connected layers with ReLU activations, trained on synthetic channel data. In contrast, five fully connected layers for the unsupervised artificial neural network (ANN) are used in^8^, which is trained offline to predict real-time phase shifts based on normalized synthetic data while maintaining target performance. Some studies propose joint optimization frameworks that integrate machine learning techniques. For example, a framework that optimizes beamforming matrices at the base station (BS) and reflective coefficients of the RIS to enhance capacity in multiuser cellular networks is introduced in^9^. Specialized DL architectures, such as “RISnet” in reference^10^, feature configurable layer numbers based on channel inputs, sharing antenna parameters, and alternating optimization between phase shifts and precoding. Additionally, deep reinforcement learning (DRL) methods are explored for beamforming approaches in both active and passive configurations. The deep deterministic policy gradient (DDPG) algorithm for the joint optimization of transmit beamforming and RIS phase shift matrices is utilized in^11^, employing actor-critic networks with tanh outputs to address imperfect CSI and hardware impairments. Reference^12^ introduces a model-free double deep Q-networks (DDQN)-based DRL approach for real-time RIS phase control, functioning in a time division duplex (TDD)- multi-input multi-output (MIMO) system without requiring individual sub-CSI.

## Related work

The recent methods for tackling channel acquisition challenges in RIS systems serving multiple users are addressed^13^. Reference^14^ discusses DL and coordinated beamforming techniques, utilizing beam prediction in MIMO networks to accommodate widely dispersed customers in the millimeter wave range. However, determining the appropriate beam formation settings requires training in MIMO arrays, which can hinder millimeter wave performance. To address these coordinated beam generation challenges, a novel machine learning-based approach was developed. This method involves multiple coordinated base stations disseminating a single uplink pilot sequence for training, enabling the intended users to convey their information (signature) and location while indicating their communication interactions with the environment. The DL approach can predict beam formation routes at base stations based on user signatures. Simulation results rely on accurate ray-tracing to achieve optimal rates, approaching the genie-aided upper bound when the optimal beam formation vectors are known with no training overhead. The RIS with a downlink millimeter wave is examined in^15^ to address positioning challenges using Fisher Information. Additionally,^16^,^17^] introduced DL methods to tackle millimeter wave issues in massive MIMO systems. However, as noted in^16^, DL can address reliability and latency challenges in predicting obstructions in millimeter waves using the MIMO approach. To mitigate training complexity in predicting the “massive MIMO” channel in millimeter wave systems,^17^ proposed an adversarial strategy to anticipate correlations between the channel and networks with new information. RIS aims to enhance wireless communication capacity^18^. However, these enhancements are usually found in conditions where the line-of-sight (LOS) link between sender and receiver is weak or completely obstructed^18^. Conventional RISs show minimal capacity improvements when the direct link is strong. The “multiplicative fading” phenomenon results in non-line-of-sight (NLOS) path loss through the RIS being significantly greater than that of the direct link^19^. This effect complicates the achievement of substantial rate gains for passive RISs across various wireless scenarios. Research on RISs has circumvented this issue by focusing on scenarios with greatly diminished direct linkages. In addition, conventional RIS systems only reflect incoming signals and cannot transmit them to users on the same side, limiting their coverage and effectiveness^20^. Deploying multiple RISs can address this coverage limitation. Recently, simultaneous transmitting and reflecting RISs (STAR-RISs) have attracted significant attention; they can reflect signals in the half-space on the same side while also transmitting them to the opposite side^20,21^. Compared to traditional RIS, STAR-RIS elements create a reconfigurable wireless environment, improving coverage for all users within a 360° ground plane.

Recent research in the field of RIS focused on enhancing networks has primarily pursued two key directions known as interference-mitigation-based (IMB)^22^ and signal-enhancement-based (SEB)^23^ designs, focusing on interference mitigation and signal enhancement, respectively. SEB design may increase interferences, hindering network performance in high signal-to-noise ratio (SNR) conditions. In low-SNR scenarios, performance remains compromised since the design fails to enhance the intended signal. Reference^24^ presents a novel simultaneous signal enhancement and cancellation method aimed at improving performance in both high and low SNR environments. STAR-RIS arrays extend signal coverage throughout space. To fully evaluate STAR-RIS performance, integrating non-orthogonal multiple access (NOMA) and STAR-RIS technology is developed to enhance connectivity and resource allocation by efficiently managing user CSI^25^. Unlike traditional RIS, which only reflects signals, the combination of STAR-RIS and NOMA in cellular networks is expected to provide comprehensive full-space communication services to a vast number of users. In this paper, a DL approach utilizing two suggested architectures of STAR-RIS is introduced. ASTAR and nearly NP-STAR hardware designs are introduced and compared. The first proposed architecture for STAR is NP-STAR, which mainly consists of passive reflecting components to reduce interference from neighboring cells while enhancing signals from the serving base station. The NP-STAR features some active elements as sensors but is primarily focused on passive operation. Furthermore, NP-STAR overcomes the limitations of traditional passive STAR-RIS by implementing configurations that enhance performance without fully adopting active components. The NP-STAR architecture integrates specific sensors capable of operating in two distinct modes: (i) channel sensing mode as channel’s descriptors, where they serve as receivers equipped with full RF chains and baseband processing capabilities, and (ii) reflection/transmission mode, where they act as passive reflecting/transmitting elements similar to other STAR-RIS components which can be managed either through individual RF chains or shared RF chain configurations, as detailed in^26^ and its references. The second proposed architecture is the sub-connected ASTAR^27^ for comparison in areas with a strong direct path to highlight its capability in addressing the “multiplicative fading” issue. It achieves this by constructing the desired signal while simultaneously canceling interference signals at the cell edge users (CEUs). Unlike passive STAR-RIS or even NP-STAR, which merely reflect signals, ASTAR incorporates active components to amplify incoming signals, where each reflecting or transmitting element is equipped with active loads, enhancing signal transmission. The proposed architecture leverages sub-connected ASTAR to optimize data transmission rates while minimizing energy consumption for receiver users. To fairly compare STAR-RIS and RIS systems benchmarks, we use the DL optimization framework from^5^, adapted for both architectures. This isolates performance gains to the STAR-RIS technology, excluding optimization algorithm differences. The adopted DL architecture, incorporating diverse network features, is designed to predict channel characteristics and dynamically adjust the STAR-RIS’s beamforming vectors in response to environmental variations. Furthermore, it ensures low training costs and minimal power consumption, delivering robust performance within the Multi-Input Single-Output (MISO) system.

### Contribution

We propose low-complexity architectures of STAR-RIS in a multiple-input single-output (MISO) downlink cellular network. The key achievements of this study are outlined in the following points:

- Our design features two STAR-RIS architectures: a sub-connected ASTAR and a low-complexity NP-STAR. The active sensors in NP-STAR act as environmental descriptors for training the DL model, while the other elements remain passive.
- Utilizing active sensors to train the proposed DL approach significantly boosts the data rate for the receiver users (CEUs). That is because these sensors serve as medium indicators, allowing the model to precisely forecast the full channel parameters of the deployed STAR-RISs while reducing training overhead. Consequently, this improves the Signal-to-Interference plus Noise Ratio (SINR) for CEUs, resulting in higher data rates.
- To obtain effective results, the proposed architectures incorporating ASTAR and NP-STAR will be compared against two benchmarks: ARIS and NP-RIS. Furthermore, evaluations will be conducted in scenarios where neither STAR nor RIS is present.
- ASTAR enhances connection quality by addressing challenges like “multiplicative fading” attenuation, leading to broader coverage and increased data rates, although NP-STAR may surpass ASTAR in certain situations.
- The DL-based downlink optimization problem is analyzed to ensure a fair comparison between the proposed STAR and RIS benchmarks in terms of data rates, spectral energy efficiency (SEE), and satisfaction rates.
- Power management control enhances transmission power at the interfering base station to constructively serve CEU2, while simultaneously reducing interference at CEU1 through destructive interference using STAR-RIS.
- We introduce the spatial temporal analysis network (STAN), as an innovative spatial optimization method designed to assess and benchmark the performance of traditional DL approach against this innovative STAN technique.

The rest of this work is arranged as follows. Section III describes the paper’s proposed system model. In addition, Section IV presents the problem formulation that includes a rate analysis based on the STAR-RIS interconnection matrix and the spectral energy efficiency analysis. Section V also proposes and discusses the methods that show how the DL system should operate. Section VI provides and examines the outcomes of the proposed DL approach to the problem of obtaining the interconnection matrix. Finally, Section VII closes and summarizes the paper’s findings.

## System model

**Fig. 1:**
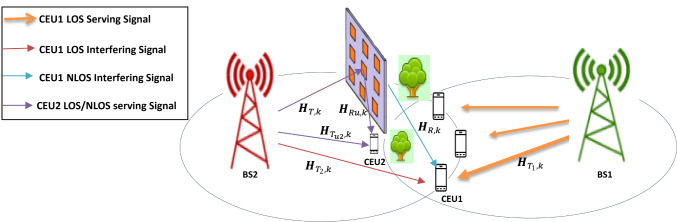
System model of a downlink wireless network.

A proposed downlink wireless network consists of two neighboring cells. The first cell is managed by a single base station, BS1, which serves as a serving BS to its users, including CEU1, located in the interference region between the two adjacent cells. The second cell is served by another base station, BS2, which provides service to CEU2 and influences as an interfering BS for CEU1, as shown in Fig. 1. The STAR-RIS is employed to counteract the interfering base station, BS2 by creating destructive interference at the CEU1, effectively mitigating the inter-cell interference (ICI) caused by the interfering BS within the overlap area of adjacent cells. Notably, the CEU is a user that receives comparatively weaker signal strength from both BSs, making it more susceptible to significant ICI from neighboring cells. Additionally, it is assumed that each BS is equipped with N and a uniform linear array (ULA) antenna elements^28^. Assume BS1 and BS2 are equipped with ${N}_{1}$ and ${N}_{2}$ antennas, respectively. While the CEU1 and CEU2 are equipped with a single antenna for both transmission and reception. Additionally, considering there is an obstacle blocking the connection between BS1 and CEU2, focus more on the CEU1 case.

In the proposed model, interference from BS2 is mitigated at CEU1, which is situated on the side of transmission using the transmit beam of a STAR-RIS, which is equipped with M elements using a uniform planar array (UPA)^29^ and positioned near BS2. Notably, there is no direct link between the STAR-RIS and BS1. CEU1 resides in the overlapping region between the two cells. As a result, CEU1 experiences ICI issues when both cells’ BSs transmit information signals simultaneously. To enhance CEU1’s performance, the STAR-RIS is effectively positioned near the BS2. Its purpose is to mitigate the ICI directed at CEU1 by adjusting the STAR-RIS’s transmitting phase shifts. The transmitted beam from the STAR-RIS is designed to interact destructively with the direct path (LOS) signals originating from the BS2 to CEU1. Meanwhile, the reflected beam of the STAR-RIS is utilized to constructively enhance the signal received from CEU2’s serving base station, BS2, which is situated on the reflection side, as can be shown in Fig. 1.

Considering an orthogonal frequency division multiplexing (OFDM) system with K subcarriers $k\in \left\{\text{1,2},3,\dots , K\right\}$ and varying candidates’ locations for the receiving CEU1, the received signal at the kth subcarrier and CEU1 is described as follows:

1 $$y_{1,k} = {\varvec{H}}_{{T_{1} ,k}}^{T} {\varvec{w}}_{{T_{1} ,k }} x_{{T_{1} k}} + \left( {{\varvec{H}}_{{T_{2} ,k}}^{T} + \varvec{ H}_{R,k}^{T} {{\boldsymbol{\uppsi}}}_{t, k} \varvec{ H}_{T,k } } \right) {\varvec{w}}_{{T_{2} ,k }} x_{{T_{2} k}} + n_{k} ,$$

where ${{\varvec{H}}}_{{T}_{1},k }\in {\mathbb{C}}^{{{\varvec{N}}}_{1}\times 1}$, and ${{\varvec{H}}}_{{T}_{2},k }\in {\mathbb{C}}^{{{\varvec{N}}}_{2}\times 1}$ indicate the small-scale fast-fading direct channel matrix from the serving BS to the CEU1 at subcarrier $k$, and the small-scale fast-fading direct channel matrix from the interfering BS to the CEU1 at subcarrier $k$. While, ${{\varvec{H}}}_{R,k }\in {\mathbb{C}}^{\text{M}\times 1}$, and ${{\varvec{H}}}_{T,k }\in {\mathbb{C}}^{\text{M}\times {N}_{2}}$ represent the transmitted downlink channel matrix from STAR-RIS to CEU1, and the uplink channel matrix from the interfering BS to the STAR-RIS, respectively.

The beamforming vectors, ${{\varvec{w}}}_{{T}_{1},k }\in {\mathbb{C}}^{{{\varvec{N}}}_{1}\times 1 }\text{ and }{{\varvec{w}}}_{{T}_{2},k }\in {\mathbb{C}}^{{{\varvec{N}}}_{2}\times 1 }$ are employed by the serving and interfering BSs to transmit the LOS useful signal ${x}_{{T}_{1}k }\in {\mathbb{C}}$ by BS1 and the interfering signal ${x}_{{T}_{2}k }\in {\mathbb{C}}$ by BS2, to CEU1 over the k^th^ subcarrier, respectively. The signal transmitted over the k^th^ subcarrier adheres to the per-subcarrier power limit $E\left[ {\left| {x_{k} } \right|^{2} } \right] = \frac{{P_{T} }}{k}$, where ${P}_{T}$ is the total transmitted power, assuming ${P}_{T}$ either equals P_1_ or P_2,_ Which are the powers from BS1 and BS2, respectively. The received noise, ${n}_{k}\sim {\mathcal{N}}_{\mathbb{C}}(0, {\sigma }_{n}^{2})$ is additive white Gaussian noise characterized by zero mean and variance ${\sigma }_{n}^{2}$.

The diagonal matrix ${{\boldsymbol{\uppsi}}}_{t,k}\in {\mathbb{C}}^{M\times M}$ referred to as the STAR-RIS interconnection transmitting matrix. It represents the interaction of the STAR-RIS with the BS2 on one side and the CEU1 on the opposite side, which is the transmission side for STAR-RIS. Where ${{\boldsymbol{\uppsi}}}_{t,k}$ in the expression ${{\varvec{H}}}_{R,k}^{T} {{\boldsymbol{\uppsi}}}_{t,k} {{\varvec{H}}}_{T,k}$ is the diagonal interconnection matrix, whose inputs are represented as a vector ${{\varvec{\psi}}}_{t,k }\in {\mathbb{C}}^{{\varvec{M}}\times 1}$, ${{\boldsymbol{\uppsi}}}_{t,k}=diag ({\beta }_{t,m}{{\varvec{\psi}}}_{t,k }) \forall m\in \text{M}$, with ${{\boldsymbol{\uppsi}}}_{t,k} = diag\left\{ {\beta_{t,1} e^{{j\emptyset_{t,1} }} ,\beta_{t,2} e^{{j\emptyset_{t,2} }} , \ldots ,\beta_{t,M} e^{{j\emptyset_{t,M} }} } \right\} \in {\mathbb{C}}^{M \times M}$, representing the STAR-RIS’s diagonal phase-shift matrix of transmission.

By the same method, the received signal at the CEU2 is:

2 $${y}_{2,k}=\left({{\varvec{H}}}_{{T}_{u2},k}^{T} +{\boldsymbol{ }{\varvec{H}}}_{Ru,k}^{T} {{\boldsymbol{\uppsi}}}_{r, k}\boldsymbol{ }{{\varvec{H}}}_{T,k }\right){ {{\varvec{w}}}_{{T}_{u2},k }x}_{{T}_{2}k}+{n}_{k},$$

where $\left({{\varvec{H}}}_{{T}_{u2},k}^{T} +{\boldsymbol{ }{\varvec{H}}}_{Ru,k}^{T} {{\boldsymbol{\uppsi}}}_{r, k}\boldsymbol{ }{{\varvec{H}}}_{T,k }\right){ {{\varvec{w}}}_{{T}_{u2},k }x}_{{T}_{2}k}$ is as $\left({{\varvec{H}}}_{{T}_{2},k}^{T} +{\boldsymbol{ }{\varvec{H}}}_{R,k}^{T} {{\boldsymbol{\uppsi}}}_{t, k}\boldsymbol{ }{{\varvec{H}}}_{T,k }\right){ {{\varvec{w}}}_{{T}_{2},k }x}_{{T}_{2}k}$ in Eq. (1) from BS2, and from reflected beam of STAR-RIS but toward the CEU2. Whereas, the reflection phase shift is ${{\boldsymbol{\uppsi}}}_{r,k}=diag \left({\beta }_{r,m}{{\varvec{\psi}}}_{r,k }\right) \forall m\in \{1, 2, \dots ,\text{M}\}$, with ${{\boldsymbol{\uppsi}}}_{r,k} = diag\left\{ {\beta_{r,1} e^{{j\emptyset_{r,1} }} ,\beta_{r,2} e^{{j\emptyset_{r,2} }} , \ldots , \beta_{r,M} e^{{j\emptyset_{r,M} }} } \right\} \in {\mathbb{C}}^{M \times M}$. Moreover, the transmitted and reflected amplitude coefficients of the m^th^ STAR-RIS element are denoted as ${\beta }_{t,m}$ ∈ [0, 1] and ${\beta }_{r,m}$ ∈ (0, 1], respectively, as in^26^. According to the energy conservation law, the combined power of the reflection and transmission coefficients satisfies: ${{\beta }_{t,m}}^{2}+ {{\beta }_{r,m}}^{2}\le 1$. For simplicity, we assume ${{\beta }_{t,m}}^{2}$+ ${{\beta }_{r,m}}^{2}$  = 1 as adopted in^26^. The phase shift coefficients for the mth element of STAR-RIS for transmission and reflection are denoted as $\emptyset_{t,m} \in \left[ {0, 2\pi } \right],\; {\text{and}}\; \emptyset_{r,m} \in \left[ {0, 2\pi } \right]$. This diagonal represents the STAR-RIS transmission process and reflection process, where each element $m \in \left\{1, 2, 3,\dots , M\right\}$ redirects its incident signal by multiplying it by an interconnection factor ${{[{\varvec{\psi}}}_{t,k }]}_{m} \forall m,k$ for transmission, and ${{[{\varvec{\psi}}}_{r,k }]}_{m} \forall m,k$ for reflection, typically involving variable magnitudes (amplification or attenuation gains). Assuming phase shifters are exclusively employed to construct STAR-RIS elements, each interconnection factor can be simplified to a phase shifter, expressed as $[{\varvec{\psi}}_{t} ]_{m} = e^{{j\emptyset_{t,m} }}$, and $[{\varvec{\psi}}_{r} ]_{m} = e^{{j\emptyset_{r,m} }}$. Since these phase shifters operate using RF circuits in the analog domain, all subcarrier signals experience the same phase shift, i.e., ${{\varvec{\psi}}}_{t,k }={{\varvec{\psi}}}_{t }, \, \text{and} \, {{\varvec{\psi}}}_{r,k }={{\varvec{\psi}}}_{r } \forall k$. These phase shifters can only direct the signal at discrete angles. To address this limitation, a pre-defined codebook ${\varvec{\mathcal{P}}}$ determines the interconnection vector or the redirecting beamforming vector ${\varvec{\psi}}\supset \{{{{\varvec{\psi}}}_{t},\boldsymbol{ }{\varvec{\psi}}}_{r}\}$. Discrete phase shifters are used to apply each selection from the beamforming codebook to transmit signals in ${\varvec{\mathcal{P}}}$.

At the subcarrier $k$, the SINR (${\Upsilon}_{1,k}$) at CEU1 can be stated as in^5^:

3 $${\Upsilon}_{1,k}=\frac{{\left|{{\varvec{H}}}_{{T}_{1},k}^{T} {{\varvec{w}}}_{{T}_{1},k }\right|}^{2}}{|{\left({{\varvec{H}}}_{{T}_{2},k}^{T} +{{\varvec{H}}}_{R,k}^{T} {{\boldsymbol{\uppsi}}}_{t, k}{{\varvec{H}}}_{T,k }\right) {{\varvec{w}}}_{{T}_{2},k }|}^{2}+{\sigma }_{n}^{2}}.$$

Hence, the SINR (${\Upsilon}_{2,k}$) at CEU2 is:

4 $${\Upsilon}_{2,k}=\frac{|{\left({{\varvec{H}}}_{{T}_{u2},k}^{T} +{\boldsymbol{ }{\varvec{H}}}_{Ru,k}^{T} {{\boldsymbol{\uppsi}}}_{r, k}\boldsymbol{ }{{\varvec{H}}}_{T,k }\right) {{\varvec{w}}}_{{Tu}_{2},k }|}^{2}}{{\sigma }_{n}^{2}}.$$

In general form, the achievable data rate can be expressed as follows:

5 $$R=\frac{1}{K}\sum_{k=1}^{K}{log}_{2}\left(1+{\Upsilon}_{k}\right),$$

i.e., the achievable data rate at CEU1 can be expressed as:

6 $${R}_{1}=\frac{1}{K}\sum_{k=1}^{K}{log}_{2}(1+\frac{{\left|{{\varvec{H}}}_{{T}_{1},k}^{T} {{\varvec{w}}}_{{T}_{1},k }\right|}^{2}}{|{\left({{\varvec{H}}}_{{T}_{2},k}^{T} +{\boldsymbol{ }{\varvec{H}}}_{R,k}^{T} {{\boldsymbol{\uppsi}}}_{t, k}\boldsymbol{ }{{\varvec{H}}}_{T,k }\right) {{\varvec{w}}}_{{T}_{2},k }|}^{2}+{\sigma }_{n}^{2}}).$$

While the achievable data rate at CEU2 will be as follows:

7 $${R}_{2}=\frac{1}{K}\sum_{k=1}^{K}{log}_{2}(1+\frac{|{\left({{\varvec{H}}}_{{T}_{u2},k}^{T} +{{\varvec{H}}}_{Ru,k}^{T} {{\boldsymbol{\uppsi}}}_{r, k}{{\varvec{H}}}_{T,k }\right) {{\varvec{w}}}_{{Tu}_{2},k }|}^{2}}{{\sigma }_{n}^{2}}).$$

## Problem formulation

### Achievable data rate

This paper seeks to enhance the CEU1’s data rate by optimizing the active beamforming weights, ${{\varvec{w}}}_{{T}_{1} }, and{\boldsymbol{ }{\varvec{w}}}_{{T}_{2}}$ at the BS1 and BS2, respectively. It also aims to determine the perfect vector of the passive beamforming for transmission ${{\varvec{\psi}}}_{t}$ at the STAR-RIS, subject to total power constraints by adjusting P_2_ to be minimum to achieve the minimum acceptable threshold data rate at the CEUs in cell 2 (CEU2). To meet this minimum threshold data rate for CEU2 despite minimizing the BS2 transmit power, the DL is used to optimize the STAR-RIS reflection beam to enhance the CEU2 signal strength by making a constructive interference between the BS2 direct link and the STAR-RIS reflected beam. Meanwhile, at CEU1, the interference is eliminated when the power of BS2 is minimized and the transmit beam in STAR-RIS is utilized to destroy the interference from BS2 at CEU1 by employing the splitting power mode over STAR-RIS. To determine the optimal redirection vectors for reflection and transmission at the STAR-RIS, we find the relations of ${{\varvec{\psi}}}_{t}^{*}$, and ${{\varvec{\psi}}}_{r}^{*}$ at CEU1 and CEU2, respectively, while adhering to the overall power constraints and all fundamental assumptions associated with STAR-RIS functionality.

8 $${{\varvec{\psi}}}_{t}^{*}=\text{arg}\underset{\boldsymbol{\psi \epsilon }{\varvec{\mathcal{P}}}}{\text{max}}\sum_{k=1}^{K}{log}_{2}(1+\frac{{\left|{{\varvec{H}}}_{{T}_{1},k}^{T} {{\varvec{w}}}_{{T}_{1},k }\right|}^{2}}{|{\left({{\varvec{H}}}_{{T}_{2},k}^{T} +{\boldsymbol{ }{\varvec{H}}}_{R,k}^{T} {{\boldsymbol{\uppsi}}}_{t, k}\boldsymbol{ }{{\varvec{H}}}_{T,k }\right) {{\varvec{w}}}_{{T}_{2},k }|}^{2}+{\sigma }_{n}^{2}}).$$

To attain the optimal data rate at CEU1, ${{\varvec{R}}}_{1}^{*}$ is defined as:

9 $${{\varvec{R}}}_{1}^{*}=\underset{{\varvec{\psi}}{\varvec{\epsilon}}{\varvec{\mathcal{P}}}}{\text{max}}\frac{1}{K}\sum_{k=1}^{K}{log}_{2}(1+\frac{{\left|{{\varvec{H}}}_{{T}_{1},k}^{T} {{\varvec{w}}}_{{T}_{1},k }\right|}^{2}}{|{\left({{\varvec{H}}}_{{T}_{2},k}^{T} +{\boldsymbol{ }{\varvec{H}}}_{R,k}^{T} {{\boldsymbol{\uppsi}}}_{t, k}\boldsymbol{ }{{\varvec{H}}}_{T,k }\right) {{\varvec{w}}}_{{T}_{2},k }|}^{2}+{\sigma }_{n}^{2}}).$$

Also, at CEU2:

10 $${{\varvec{\psi}}}_{r}^{*}=\text{arg}\underset{\boldsymbol{\psi \epsilon }{\varvec{\mathcal{P}}}}{\text{max}} \sum_{k=1}^{K}{\mathit{log}}_{2}(1+\frac{|{\left({{\varvec{H}}}_{{T}_{u2},k}^{T} +{\boldsymbol{ }{\varvec{H}}}_{\mathit{Ru},k}^{T} {{\boldsymbol{\uppsi}}}_{r, k}\boldsymbol{ }{{\varvec{H}}}_{T,k }\right) {{\varvec{w}}}_{{\mathit{Tu}}_{2},k }|}^{2}}{{\sigma }_{n}^{2}}),$$

And the optimal data rate, ${{\varvec{R}}}_{2}^{*}$ at CEU2, is defined as:

11 $${{\varvec{R}}}_{2}^{*}=\underset{{\varvec{\psi}}{\varvec{\epsilon}}{\varvec{\mathcal{P}}}}{\text{max}}\frac{1}{K}\sum_{k=1}^{K}{log}_{2}(1+\frac{|{\left({{\varvec{H}}}_{{T}_{u2},k}^{T} +{\boldsymbol{ }{\varvec{H}}}_{Ru,k}^{T} {{\boldsymbol{\uppsi}}}_{r, k}\boldsymbol{ }{{\varvec{H}}}_{T,k }\right) {{\varvec{w}}}_{{Tu}_{2},k }|}^{2}}{{\sigma }_{n}^{2}}).$$

The optimization problem, including both ${{\varvec{\psi}}}_{t}^{*}$, and ${{\varvec{\psi}}}_{r}^{*}$ lacks a closed-form solution, posing a significant challenge in determining the optimal STAR-RIS interconnection vector of transmission ${{\varvec{\psi}}}_{t}^{*}$ and of reflection ${{\varvec{\psi}}}_{r}^{*}$ that provide the optimum rates ${{\varvec{R}}}_{1}^{*},$ and ${{\varvec{R}}}_{2}^{*}$ without requiring exhaustive searches through the predefined codebook ${\varvec{\mathcal{P}}}$.

Following the methodology and formulas outlined in^27^, the optimal redirection vectors for reflection and transmission when employing ASTAR in place of NP-STAR are derived in^27^ as follows:

At CEU1:

12 $${{\varvec{\psi}}}_{t}^{*}=\text{arg}\underset{\boldsymbol{\psi \epsilon }{\varvec{\mathcal{P}}}}{\text{max}}\sum_{k=1}^{K}{log}_{2}(1+\frac{{\left|{{\varvec{H}}}_{{T}_{1},k}^{T} {{\varvec{w}}}_{{T}_{1},k }\right|}^{2}}{{\left|\left({{\varvec{H}}}_{{T}_{2},k}^{T} +{\boldsymbol{ }{\varvec{H}}}_{R,k}^{T} {{\boldsymbol{\uppsi}}}_{t, k}\boldsymbol{ }{{\varvec{H}}}_{T,k }\right) {{\varvec{w}}}_{{T}_{2},k }\right|}^{2}+{\Vert {\boldsymbol{ }{\varvec{H}}}_{R,k}^{T} {{\boldsymbol{\uppsi}}}_{t, k}\Vert }^{2}{\sigma }_{z}^{2}+{\sigma }_{n}^{2}}),$$

And the optimal data rate at CEU1, ${{\varvec{R}}}_{1}^{*}$ is defined as:

13 $${{\varvec{R}}}_{1}^{*}=\underset{{\varvec{\psi}}{\varvec{\epsilon}}{\varvec{\mathcal{P}}}}{\text{max}}\frac{1}{K}\sum_{k=1}^{K}{log}_{2}(1+\frac{{\left|{{\varvec{H}}}_{{T}_{1},k}^{T} {{\varvec{w}}}_{{T}_{1},k }\right|}^{2}}{{\left|\left({{\varvec{H}}}_{{T}_{2},k}^{T} +{\boldsymbol{ }{\varvec{H}}}_{R,k}^{T} {{\boldsymbol{\uppsi}}}_{t, k}\boldsymbol{ }{{\varvec{H}}}_{T,k }\right) {{\varvec{w}}}_{{T}_{2},k }\right|}^{2}+{\Vert {\boldsymbol{ }{\varvec{H}}}_{R,k}^{T} {{\boldsymbol{\uppsi}}}_{t, k}\Vert }^{2}{\sigma }_{z}^{2}+{\sigma }_{n}^{2}}).$$

Also, at CEU2:

14 $${{\varvec{\psi}}}_{r}^{*}=\text{arg}\underset{\boldsymbol{\psi \epsilon }{\varvec{\mathcal{P}}}}{\text{max}} \sum_{k=1}^{K}{\mathit{log}}_{2}(1+\frac{{\left|\left({{\varvec{H}}}_{{T}_{u2},k}^{T} +{\boldsymbol{ }{\varvec{H}}}_{\mathit{Ru},k}^{T} {{\boldsymbol{\uppsi}}}_{r, k}\boldsymbol{ }{{\varvec{H}}}_{T,k }\right) {{\varvec{w}}}_{{\mathit{Tu}}_{2},k }\right|}^{2}}{{\Vert {\boldsymbol{ }{\varvec{H}}}_{\mathit{Ru},k}^{T} {{\boldsymbol{\uppsi}}}_{r, k}\Vert }^{2}{\sigma }_{z}^{2}+{\sigma }_{n}^{2}}),$$

and ${{\varvec{R}}}_{2}^{*}$ at CEU2, is defined as:

15 $${{\varvec{R}}}_{2}^{*}=\underset{{\varvec{\psi}}{\varvec{\epsilon}}{\varvec{\mathcal{P}}}}{\text{max}}\frac{1}{K}\sum_{k=1}^{K}{log}_{2}(1+\frac{{\left|\left({{\varvec{H}}}_{{T}_{u2},k}^{T} +{\boldsymbol{ }{\varvec{H}}}_{Ru,k}^{T} {{\boldsymbol{\uppsi}}}_{r, k}\boldsymbol{ }{{\varvec{H}}}_{T,k }\right) {{\varvec{w}}}_{{Tu}_{2},k }\right|}^{2}}{{\Vert {\boldsymbol{ }{\varvec{H}}}_{Ru,k}^{T} {{\boldsymbol{\uppsi}}}_{r, k}\Vert }^{2}{\sigma }_{z}^{2}+{\sigma }_{n}^{2}}).$$

The noise terms at ASTAR are ${{\varvec{H}}}_{R,k}^{T}{{\boldsymbol{\uppsi}}}_{t,k}{\varvec{z}}$ and ${{\varvec{H}}}_{Ru,k}^{T}{{\boldsymbol{\uppsi}}}_{r,k}{\varvec{z}}$ in most cases of passive and NP-STAR-RIS, can be disregarded; where ${\varvec{z}}\boldsymbol{ }\sim { \mathcal{N}}_{\mathbb{C}}(0, {\sigma }_{z}^{2})$ is the ASTAR thermal noise; however, for ASTAR, both the noise at the ASTAR and the incoming signal are amplified, making the terms “${{\varvec{H}}}_{R,k}^{T} {{\boldsymbol{\uppsi}}}_{t, k}{\varvec{z}}$” and “${{\varvec{H}}}_{Ru,k}^{T}{{\boldsymbol{\uppsi}}}_{r,k}{\varvec{z}}$” significant and impossible to ignore.

### Spectral energy efficiency

Spectral energy efficiency (SEE) is a critical parameter in the design of STAR-RIS technology and serves as a key performance indicator for enabling efficient and green wireless networks^30^. STAR-RIS technology transmits and reflects the received signals by leveraging an optimized phase shift design for its reflective/transmissive elements, ensuring a constructive signal combination for transmission and reflection without relying on dedicated power amplifiers. Unlike traditional Amplify-and-Forward (AF) relays, NP-STAR operates without amplifiers, resulting in significantly lower energy consumption. On the opposite side, the sub-connected ASTAR is utilized to mitigate the high power consumption associated with the fully-connected ASTAR. The overall consumed power ${P}_{C}$ for the proposed NP-STAR structure used to evaluate energy efficiency (EE) as outlined in^5,27^,^31^. It can be generally expressed as follows:

16 $${P}_{C}=\varepsilon \sum_{k=1}^{K}{\Vert {{\varvec{w}}}_{{T}_{1},k}\Vert }^{2}+ M{P}_{PS}+{ \overline{M } (P}_{LNA}+{P}_{RFchain}+{2P}_{ADC})+{P}_{BB}+{P}_{BS}+{K}_{u}{P}_{UE}.$$

The terms $\varepsilon ,{P}_{PS}$*, *${P}_{LNA}$*, *${P}_{RFchain}$*,* ${\text{P}}_{\text{BB}}$, and ${\text{P}}_{\text{BS}}$ represent the reversal of energy conversion coefficients at the BS1, the static power consumed in the phase-shift circuit, the low noise amplifier (LNA), RF chains, the baseband processor, and the oscillator and circuit at the BS, respectively. While ${K}_{u}, and {P}_{UE}$ is the users’ number and the user circuit power consumption. Additionally, the “Analog to Digital Converter” (ADC) power consumption is denoted as ${P}_{ADC}$ can be expressed as:

17 $${P}_{ADC}={FOM}_{W}\times {f}_{s}\times {2}^{n}.$$

The figure-of-merit for walden (${FOM}_{W}$) is used to evaluate the power efficiency ranking of the ADC^32^,^33^, where ${f}_{s}$ represents the Nyquist sampling frequency.

In addition, the total consumed power for the proposed sub-connected ASTAR structure ${P}_{C, active}$ can be calculated^27^, as follows:

18 $$\begin{aligned} P_{C, active} & = \varepsilon \mathop \sum \limits_{k = 1}^{K} {\varvec{w}}_{{T_{1} ,k}}^{2} + {\varvec{\zeta}}\left( {\mathop \sum \limits_{{{\varvec{k}} = 1}}^{{\varvec{K}}} \left( {\left\| {{\varvec{\psi}}_{{{\varvec{t}},\varvec{ k}}} \varvec{ H}_{{{\varvec{T}},\varvec{k }}} {\varvec{w}}_{{{\varvec{T}}_{2} ,\varvec{i }}} } \right\|^{2} + \left\| {{\varvec{\psi}}_{{{\varvec{r}},\varvec{ k}}} \varvec{ H}_{{{\varvec{T}},\varvec{k }}} {\varvec{w}}_{{{\varvec{Tu}}_{2} ,\varvec{k }}} } \right\|^{2} + \left( {\left\| {{\varvec{\psi}}_{{{\varvec{t}},\varvec{ k}}} } \right\|^{2} + \left\| {{\varvec{\psi}}_{{{\varvec{r}},\varvec{ k}}} } \right\|^{2} } \right)\sigma_{{\varvec{z}}}^{2} } \right)} \right) \\ & \quad + LW_{PA} + MP_{PS} + P_{BB} + P_{BS} + K_{u} P_{UE} . \\ \end{aligned}$$

where ${\varvec{\zeta}}$, and ${W}_{PA}$ represent the reversal of energy conversion coefficients at ASTAR and the static power of the sub-connected ASTAR hardware’s power amplifiers, respectively, where $L=\frac{M}{T}$ , indicates the number of amplifiers used, each supplying $T$ ASTAR elements in the sub-connected design.

Lastly, ${\eta }_{EE}$ is the spectral energy efficiency SEE which is expressed using the following equation:

19 $${\eta }_{EE}\left(\frac{bits}{joule}\right)=\frac{R\times W}{{P}_{C}},$$

where $R$ represents the achievable data rate, while W denotes the transmitted channel bandwidth.

## Methods

**Fig. 2:**
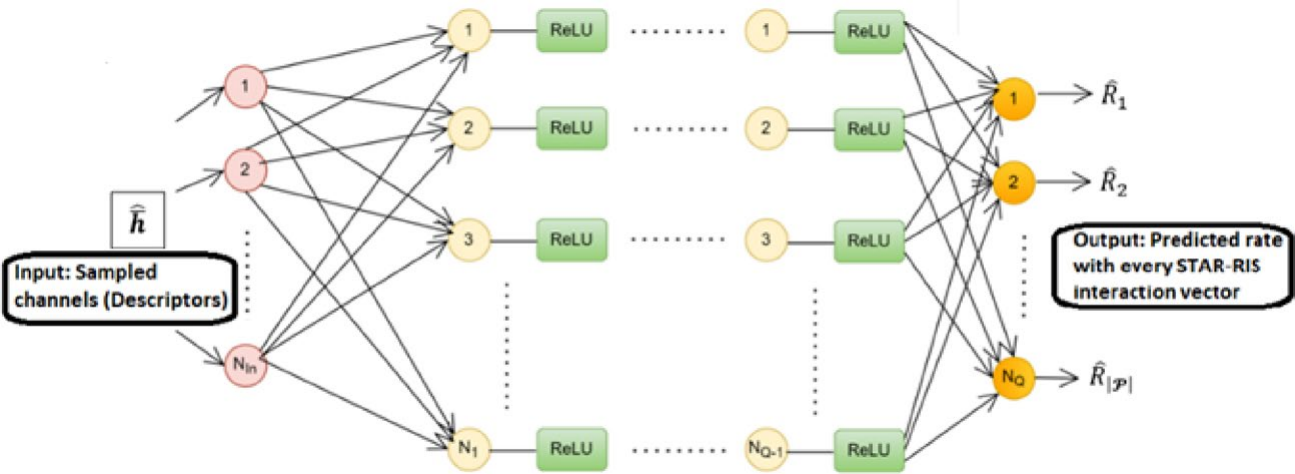
The adopted neural network design includes fully connected layers, each followed by a non-linear ReLU activation function^34^.

This paper employs a DL technique to enhance the achievable data rate of the CEU1 and CEU2, which relies on the channel matrices by utilizing select active elements (sensors) of the STAR-RIS. These sensors serve as indicators of the observed channels, providing insights into the multipath characteristics. Once environmental information is collected through these indicators, a DL algorithm predicts the optimal STAR-RIS interconnection (redirecting) vectors (for transmission and reflection). The DL algorithm aims to identify a functional relationship that maps the indicator vector to the STAR-RIS interconnection vectors (reflection/transmission beamforming)^34^, as illustrated in Fig. 2.

The main challenge of the STAR-RIS system lies in the high hardware and training costs associated with developing the STAR-RIS connectivity (reflection/transmission beamforming) vectors. To tackle this problem and simplify the STAR-RIS system, the architecture will utilize randomly selected active elements, such as sensors. As illustrated in Fig. 1, the NP-STAR comprises $\text{M}$ passive reflecting/transmitting elements with $\overline{\text{M} }$ (M_bar) active reflecting elements, where $\overline{\text{M} }<\text{M}$. The passive elements, which are not connected to the baseband unit, are implemented by RF phase shifters. The $\overline{\text{M} }$ active reflectors are randomly chosen from the passive elements and can operate in two modes: first, as receivers with full RF chains and baseband processing for channel sensing; second, as passive elements reflecting/transmitting the incident signal in reflection/transmission mode. The same method will be followed when replacing the NP-STAR with ASTAR, but the ASTAR’s elements will be subconnected active elements as adopted in^27^. The adopted DL model, illustrated in Fig. 2, learns the correlation between the observed channel vector (medium indicators) and the optimal STAR-RIS redirecting vectors. It is based on a fully connected feedforward network, also known as a multi-layer perceptron (MLP)^35^. The system features one input and one output, with the MLP comprising four hidden layers equipped with non-linear Rectified Linear Units (ReLUs) activation functions^36^ and a dropout layer set at a 0.5 rate. The output layer is fully connected to a regression layer. Training options include mini-batch size, maximum epochs, initial learning rate, learning rate schedule, L2 regularization, and validation data. A dropout layer follows each ReLU layer. Unless specified otherwise, training uses a batch size of 500, a dropout rate of 50%, ${L}_{2}$ regularization factor of 10^–4^, and 20 epochs. The learning rate starts at 0.1 and is halved every three epochs. The model is trained for 20 epochs, using validation data to monitor performance, and is then utilized to predict outputs for that data. It is important to note that the adopted DL architecture and its hyperparameters represent a conventional configuration rather than an optimized framework specifically tailored for STAR-RIS applications. These hyperparameters adhere to established practices in the literature^5,27^, ensuring methodological consistency and facilitating fair comparisons with existing RIS benchmarks.

The proposed STAR-RIS interconnection approach using DL operates in two stages. The first stage involves training, where the STAR-RIS employs an extensive look-up for redirecting beamforming to compile a dataset for the DL model. Once the dataset is fully acquired, the model training occurs, leading to the second stage, known as the anticipation stage. In this stage, the estimated channel vector $\widehat{\overline{{\varvec{h}}} }$ from the training phase is used to determine the redirecting beamforming vector, as outlined in Algorithm 1.

The STAR-RIS interconnection vector can be designed using observed channel vectors, ${\overline{{\varvec{H}}} }_{T,k}$**,** ${\overline{{\varvec{H}}} }_{R,k}$, which can be determined by transmitting uplink training single pilots from both the transmitter and receiver. This interaction maximizes engagement with all active elements to obtain the necessary observed channels. Once these channels are known, the complete channels ${{\varvec{H}}}_{T,k}$, ${{\varvec{H}}}_{R,k}$ can be predicted using the DL algorithm based on the observed indicators. Consequently, the RIS system interacts effectively with the wireless signal through a precise anticipating function that connects the medium indicators to the optimal interconnection vector. This enables the STAR-RIS to approach the best achievable data rate using the DL technique, maintaining low computational complexity and requiring only a few active RIS elements with minimal training overhead.

The computational complexity of DL methods can vary significantly based on simulation factors like the model architecture, its size, and the input data complexity. The computational complexity of the proposed structure, expressed in Big *O* notation, is given from^27^,^37^ as: $O(E\cdot b\cdot ({N}_{L}.{N}_{neurons}^{2}+{M}^{2}/T +{N}_{1}+ M{N}_{2}+ |{\varvec{\mathcal{P}}}|\cdot ({M}^{2}))$, for ASTAR, and as: $O(E\cdot b\cdot ({N}_{L}.{N}_{neurons}^{2}+{\overline{M} }^{2}+{N}_{1}+ M{N}_{2}+ |{\varvec{\mathcal{P}}}|\cdot ({\overline{M} }^{2}))$, for the NP-STAR. The term $E\cdot b$ represents the total number of training iterations in the context of DL, while ${N}_{L}.{N}_{neurons}^{2}$ reflects the computational complexity associated with processing through the layers of a neural network, where ${N}_{L}=4$. Additionally, the terms $O\left({M}^{2}/T\right), O\left({\overline{M} }^{2}\right), O({N}_{1}+ M{N}_{2}),\text{ and }O(|{\varvec{\mathcal{P}}}|\cdot ({\overline{M} }^{2}))$ represent the complexities related to ASTAR (sub-connected) processing, NP-STAR with active sensors, channel estimation, and codebook search, respectively. These limitations are necessary to avoid memory overflow and excessive runtime, given the system specifications of 64 GB RAM (dynamic), 32 logical processors, and 127 GB storage under UEFI GEN2 for Windows 10 ISO, using MATLAB 2018a. Algorithm 1 summarizes the operational framework pseudocode for the training and prediction stages of the proposed DL-based STAR-RIS redirecting (interaction) technique. The code runs in two distinct stages: (I) the training stage and (II) the prediction stage.

**Algorithm 1:**
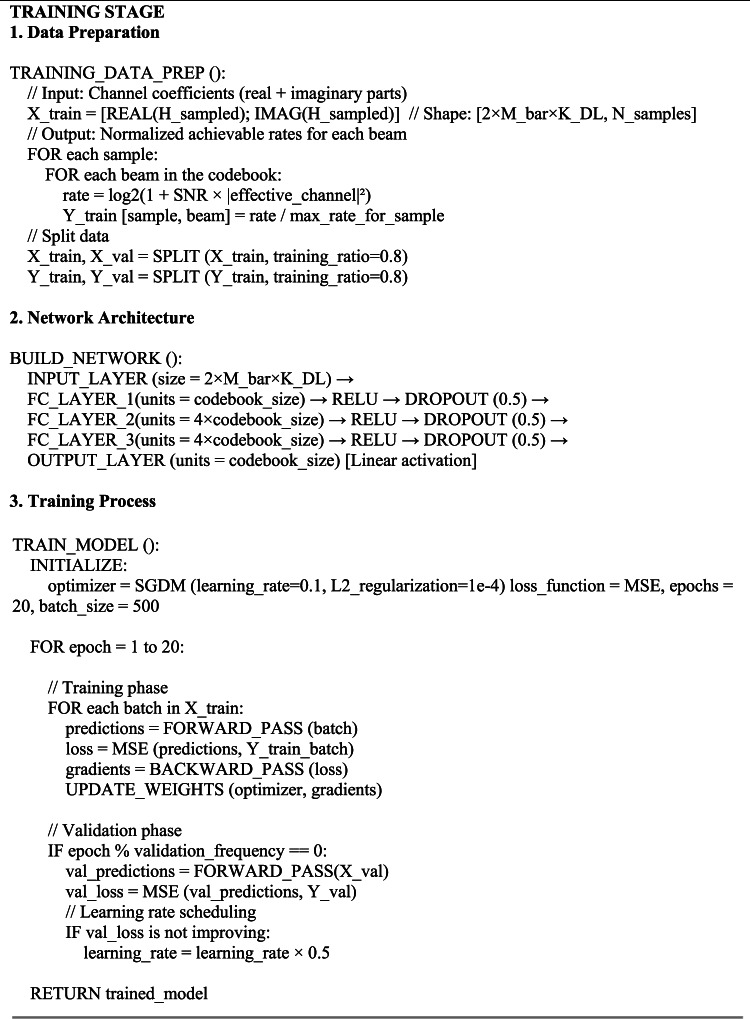

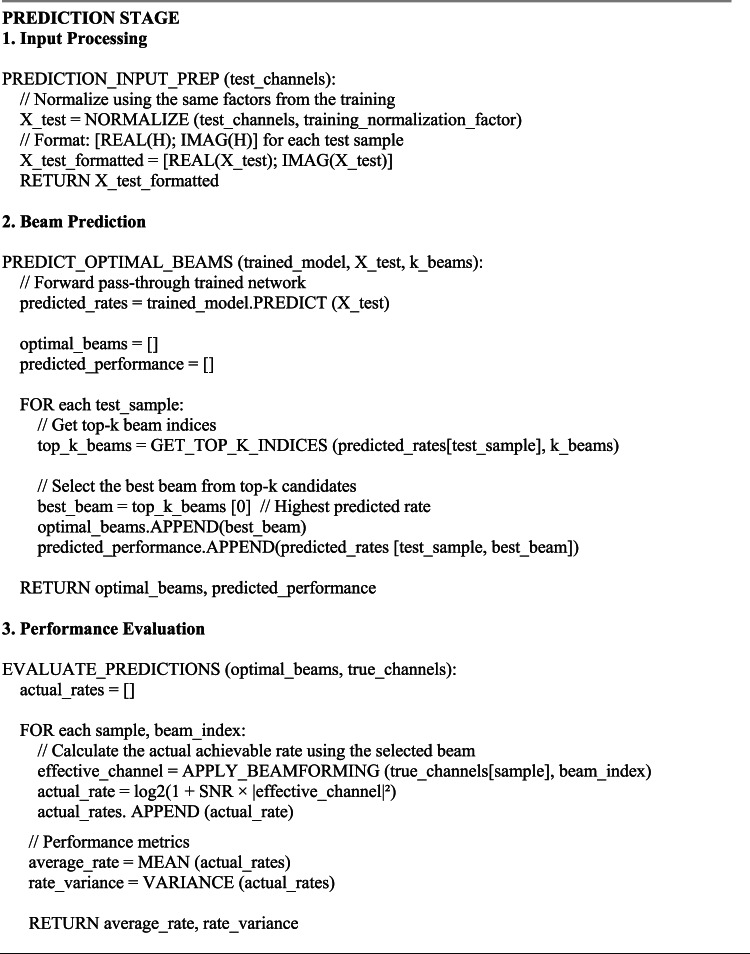
DL training and prediction stages - pseudocode.

The results presented in this paper were achieved using the publicly available DeepMIMO dataset for the "ray-tracing O1" scenario, designed for outdoor environments^38,39^.

**Fig. 3:**
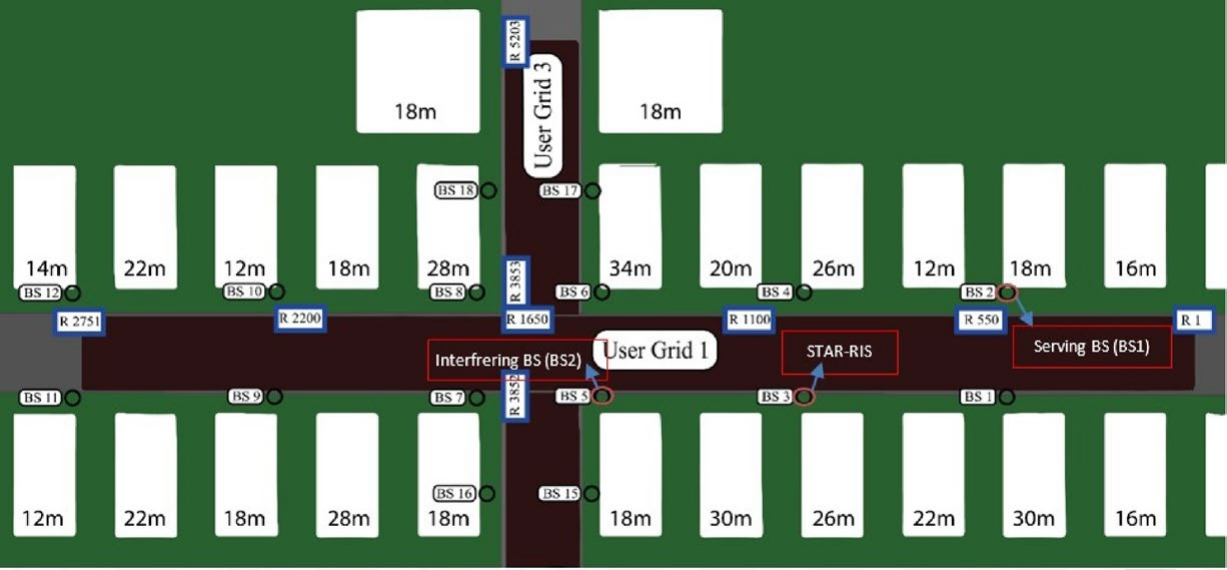
The “’ O1’ scenario” created by “Remcom Wireless InSite” referenced in^38^,^39^].

**Fig. 4:**
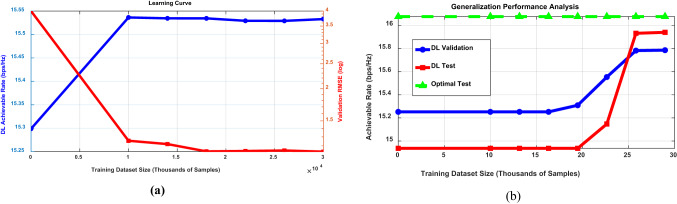
Dataset size analysis: (**a**) Learning curve validation and (**b**) generalization performance comparison across training, validation, and test sets.

This dataset, developed with “Remcom Wireless InSite,” depicts a setting with two intersecting roads, as illustrated in Fig. 3. At the intersection of these streets surrounded by buildings, there are 18 base stations (BSs) and x-y grids representing user locations. In the proposed setup, the STAR-RIS is situated close to BS 5. Furthermore, BS 2 serves the users, while BS 5 interferes. The CEU1 receiver positions are arranged in 200 rows (R800-R1000), and also, CEU2 in 200 rows (R1020-R1220), each containing 181 points. For training and validation, we use 36,200 data points from the DeepMIMO dataset, split into 83% for training and 17% for validation. This strategy was designed to optimize training efficiency while focusing on model development as can be shown in Fig. 4. As shown in Fig. 4a, the intersection point between 5000 and 10,000 samples highlights the optimal balance between training efficiency and generalization capability. Based on this observation, we initially selected a training set of 30,000 samples (ensuring sufficient data beyond the saturation point) and a validation set of 6,200 samples (providing robust performance evaluation). This 83–17% split aimed to ensure stable model performance while allocating adequate data for validation, as evidenced by consistently low validation RMSE beyond the 10,000-sample threshold.

Figure 4b alleviates generalization concerns by illustrating a strong alignment between validation and test performance across various training dataset sizes. The Fig. 4b results clearly demonstrate that both validation and test performance steadily improved with the increasing size of the training dataset, with no signs of overfitting. Notably, the DL model’s test performance nears the optimal benchmark, highlighting its strong generalization ability beyond the training data.

## Results and discussion

**Fig. 5:**
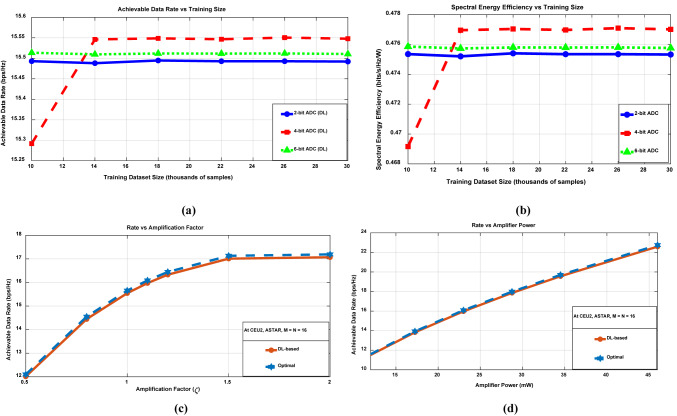
Resolution sensitivity analysis: (**a**) ADC achievable data rate. (**b**) ADC spectral energy efficiency versus DL training dataset size. (**c**) ASTAR rate versus amplification factor. (**d**) ASTAR rate versus amplifier power.

This subsection assesses the potential data rate and SEE of STAR-RIS systems utilizing DL-assisted reflection (redirecting) beamforming, as outlined in rate Eqs. (9) and (11), and the SEE Eq. (19). It is worth mentioning that the Genie-Aided Reflection Beamforming acts as the upper-performance benchmark, operating with perfect CSI knowledge. In addition, the SEE is analyzed in Bits/Joule, considering variations in the number of $\overline{M }$ (channel sensors) and $M$ of the STAR-RIS, respectively. The proposed system model assumes $\overline{M }$ active elements connected via a fully digital architecture to the baseband, utilizing ADCs with n-bit resolution. The parameters adopted for this analysis are as follows: ${P}_{LNA}=20 mW$, ${P}_{PS}=10 mW$, ${\text{P}}_{\text{BB}}=200 mW$, and ${P}_{RFchain}=40 mW$ with a bandwidth of $W=100 MHz$^31^. Additionally, the ${FOM}_{W}$ is assumed to be $46.1$ fJ/conversion-step as referenced in^34^,^40^. The ADC resolution is set to $n=4$ bits, as determined by the trade-off outlined in^32^ for a fully digital architecture between power consumption and the achievable data rate and as justified in Fig. 5 which analyzes our performance sensitivity. The ADC resolution sensitivity analysis is demonstrated using STAR-RIS at CEU2, with M = N = 16, and P2 = − 10 dBw as can be shown in Fig. 5a,b.

The sensitivity analysis in Fig. 5a,b confirms that selecting a 4-bit ADC resolution strikes the ideal balance, achieving optimal signal fidelity and energy efficiency, making it well-suited for practical STAR-RIS deployment. The findings indicate that higher-resolution ADCs (6-bit) lead to diminished performance due to excessive power consumption without corresponding rate improvements, while lower-resolution ADCs (2-bit) lack sufficient quantization accuracy, compromising performance. The sensitivity analysis for the STAR-RIS presented in Fig. 5c,d provides critical insights into active STAR-RIS optimization. Figure 5c highlights that both the DL-based ASTAR and the optimal approaches converge at 17 bps/Hz when ${\varvec{\zeta}}$ ≥ 1.5, with the highest sensitivity observed in the ${\varvec{\zeta}}$ range of 0.5 to 1.5. Meanwhile, Fig. 5d illustrates a nearly linear rate improvement as amplifier power increases from 12 to 46 mW, where ASTAR achieves near-optimal performance. A detailed trade-off analysis reveals diminishing returns beyond ${\varvec{\zeta}}$ = 1.5, where a 33% increase in amplification yields minimal performance gains. Additionally, power efficiency declines significantly, as achieving 23 bps/Hz demands three times the power required for 14 bps/Hz. The optimal operating point for our model is identified at ${\varvec{\zeta}}$ = 1.1 to overcome the noise amplification, with an amplifier power range of 23–30 mW, offering a balance between achievable data rate and energy efficiency.

Table 1 presents most of the DeepMIMO dataset values utilized in the simulations, while additional details can be found in references^5,35,40^. While each receiver has a single antenna with a transmit power of 5 dBw, and both transmit and receive antennas have a gain of 3 dBi. Antenna separation is 0.5 $\lambda$, where $\lambda$ is the wavelength.

**Table 1:** “DeepMIMO” dataset validated values^5,35^,^40^.

“DeepMIMO” dataset factors	Measurement
Frequency	28 GHz
Bandwidth	100 MHz
Antenna spacing	0*.*5 $\lambda$
Activated reception users	CEU1 from row R800 to R1000 CEU2 from row R1020 to R1220
Activated BSs	{2, 3, 5}
STAR-RIS antenna number $M$, and the number of antennas in BSs $N$	$\left(\text{M}\right)\in \{8;16;32;64\}$; Assuming, ${N}_{1}={N}_{2}=N=16$,
OFDM subcarriers number	512
OFDM limit (K_DL)	64

**Fig. 6:**
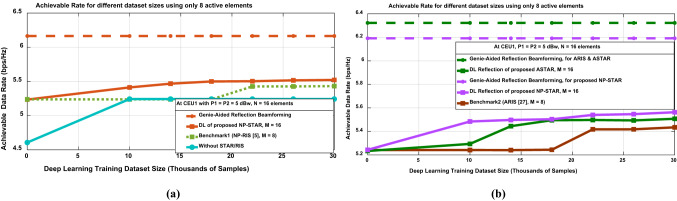
Achievable data rate at CEU1 using the STAR-RIS, RIS, and without using both of them versus DL training datasets using (**a**) nearly passive elements (NP-STAR/NP-RIS benchmark). (**b**) active elements (ASTAR/ARIS benchmark) compared to NP-STAR.

Figure 6 provides an in-depth comparison of the data rates achievable at CEU1 under various system configurations, with P_1 _= P_2 _= 5 dBw and N = 16 antenna elements, analyzing both nearly passive elements (NP-STAR/NP-RIS) and active elements (ASTAR/ARIS) under varying DL training dataset sizes.

In Fig. 6a, which focuses on nearly passive elements, the Genie-Aided Reflection Beamforming upper-performance benchmark, achieves approximately 6.2 bps/Hz. The DL Reflection Beamforming with the proposed NP-STAR shows consistent performance improvement as the training dataset increases, starting at around 5.2 bps/Hz and reaching 5.5 bps/Hz after 30,000 samples. Systems without STAR/RIS display lower initial performance, around 4.6 bps/Hz, but exhibit significant gains with training, eventually converging to about 5.23 bps/Hz. Notably, the benchmark of NP-RIS configuration^5^ with 8 elements (used to ensure a balanced comparison) achieves performance comparable to the non-STAR-RIS system after sufficient training.

In Fig. 6b, both ASTAR and NP-STAR achieve consistent Genie-Aided performance around 6.35 bps/Hz but exhibit distinct DL convergence patterns. The proposed ASTAR architecture (M = 16) reaches approximately 5.5 bps/Hz after 20,000 samples, outperforming benchmark ARIS^27^ but underperforming NP-STAR. This unexpected outcome can be attributed to two primary factors in the CEU1 environment: first, the strong direct path signal from the interfering base station at CEU1 enhances interference signals alongside the desired signal in ASTAR’s active elements. Second, the noise amplification characteristic of ASTAR’s active components worsens the situation by amplifying both thermal noise and interference signals. Conversely, the proposed NP-STAR configuration, despite its slower learning curve, demonstrates superior overall performance. Its nearly passive elements effectively minimize interference amplification, resulting in an enhanced signal-to-interference-plus-noise ratio (SINR) in high-interference scenarios. This highlights a critical design consideration: while the active elements of ASTAR generally offer greater potential gains under favorable channel conditions, their benefits can be undermined in environments with significant interference due to amplified noise and interference. Therefore, the decision between active and nearly passive implementations should be carefully assessed based on the interference characteristics of the deployment environment. The findings offer crucial insights into the complex interplay between hardware capabilities and environmental interference, highlighting that advanced hardware does not necessarily ensure superior performance under challenging interference conditions.

**Fig. 7:**
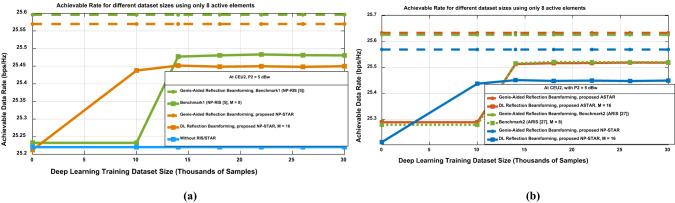
Achievable data rate at CEU2 using the STAR-RIS, RIS, and without using both of them versus DL training datasets using (**a**) nearly passive elements (NP-STAR/RIS). (**b**) active elements (ASTAR/ARIS).

Additionally, Fig. 7 provides compelling evidence of the performance benefits of DL reflection beamforming approaches at CEU2, comparing nearly passive elements (NP-STAR/RIS) with active elements (ASTAR/ARIS) across varying DL training dataset sizes. In Fig. 7a, which examines systems utilizing (M = N = 16 antenna elements), the benchmark DL Reflection Beamforming with NP-RIS^5^, using 8 elements, achieves the highest data rate of approximately 25.48 bits/Hz, closely approaching the Genie-Aided rate of about 25.6 bits/Hz with extensive training of up to 14,000 samples. In contrast, the NP-STAR configuration achieves a comparable data rate of approximately 25.45 bits/Hz but requires a smaller dataset of around 10,000 samples to converge to the Genie-Aided rate of approximately 25.57 bits/Hz.

The power splitting occurred at the STAR-RIS that enabled the NP-STAR to serve in both directions, causing this outperformance of the benchmark1 of NP-RIS^5^ over NP-STAR in the achieved data rate. Systems without RIS/STAR exhibit the lowest performance, approximately 25.22 bits/Hz, underscoring the benefits of incorporating intelligent reflecting surfaces. Moreover, Fig. 7b expands the analysis to the system with N = 16 antenna elements, revealing more significant performance gaps between the methods. The ASTAR in case of high SNR and strong direct path between BS and the receiver user with DL Reflection Beamforming for M = 16 demonstrates superior results than STAR/RIS, this is because the ASTAR can overcome the multiplicative fading effect caused by the NLOS path (BS-STAR-CEU) for such a case^27^. Where the ASTAR achieved data rates exceeding 25.52 bits/Hz with sufficient training data, which is approaching the upper bound that is fixed at 25.64 bits/Hz. This marks a notable improvement over the DL Reflection Beamforming approach at CEU2 using NP-STAR, which peaks at around 25.45 bits/Hz. The findings also highlight that active elements, coupled with larger training datasets, enable more effective optimization of ARIS configurations in^27^ with M = 8 elements; to ensure a balanced comparison, and to achieve a data rate approximately the same as ASTAR. It is worth mentioning that the effect of the power splitting that occurred in ASTAR can be overcome using the active elements, which make the ASTAR performance nearly the same as the ARIS^27^. These results carry significant implications for the design of next-generation wireless communication systems, particularly in scenarios demanding high data rates. The study clearly shows that integrating active elements with DL-based optimization can substantially enhance system performance, provided adequate training data is available. To optimize the power control on BSs’ transmit power, Table 2 illustrates the relationship between achievable data rates and DL training dataset sizes for the STAR-RIS system under varying BS2 transmit power levels, comparing performance at the CEU1 and CEU2. Table 2, which focuses on multiple performance categories that develop at different transmit power levels, demonstrates the importance of both transmit power configuration and user location regarding the STAR-RIS system. While increasing transmit power at CEU2 from its serving BS, BS2, improves overall performance, it also introduces more interference at CEU1. Thus, the relationship between BS power levels and achievable data rates is not strictly linear and varies greatly depending on user location.

**Table 2:** Achievable data rate using the STAR-RIS at different transmit power levels (P_2_) along with DL training datasets at CEU1 and CEU2.

Power Level	The achievable data rate at CEU1 (DL reflection beamforming)	The achievable data rate at CEU2 (DL reflection beamforming)
P_2_ = − 10 dBw	~ 12.5–14.7 bps/Hz	~ 15.3–15.6 bps/Hz
P_2_ = − 5 dBw	~ 10–12 bps/Hz	~ 18.4–19 bps/Hz
P_2_ = 0 dBw	~ 8.2–9.5 bps/Hz	~ 21.5–22 bps/Hz
P_2_ = 5 dBw	~ 5.3–6 bps/Hz	~ 25.5–25.8 bps/Hz

**Fig. 8:**
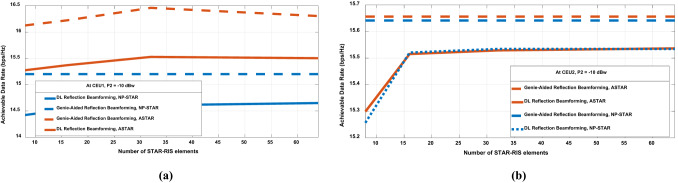
Achievable data rate comparing the ASTAR and NP-STAR at P_2_ = -10 dBw versus STAR-RIS number of elements at (**a**) CEU1. (**b**) CEU2.

Furthermore, training dataset size has a greater impact under challenging channel conditions (as in CEU1) compared to more favorable locations (as in CEU2). Furthermore, Fig. 8 compares achievable data rates of ASTAR and NP-STAR technologies based on the number of STAR-RIS elements, featuring two Figures (a) and (b), which represent measurements at CEU1 and CEU2, respectively, both at P_2_ = -10 dBw. In Fig. 8a, the DL Reflection Beamforming with ASTAR consistently outperforms NP-STAR for destructively canceling the undesired signal from BS2 at CEU1 in such low-interference scenarios. Showing a data rate increase from about 15.2 bps/Hz to 15.5 bps/Hz as the number of STAR-RIS elements rises from 8 to 32. Moreover, the improvement flattens or even decreases as it nears between 32 and 64 elements, indicating diminishing returns past a certain threshold. In contrast, NP-STAR starts at approximately 14.4 bps/Hz and levels off near 14.6 bps/Hz. The Genie-Aided Reflection Beamforming with ASTAR consistently outperforms all other approaches, maintaining around 16.5 bps/Hz, establishing an upper performance bound. In contrast, the Genie-Aided NP-STAR implementations show relatively flat performance across all element counts, with data rates stabilizing around 15.2 bps/Hz. Meanwhile, Fig. 8b shows a contrasting trend at CEU2, where ASTAR and NP-STAR attain comparable performance after around 15 STAR-RIS elements. Each technology demonstrates a significant rise in performance from 8 to 16 elements before settling at 15.52 bps/Hz, with a minor increase in achievable data rate and an increase in STAR-RIS elements from 16 to 64 elements. Notably, after 16 elements, NP-STAR marginally performs better than ASTAR, especially under low SNR conditions from BS2. This performance advantage occurs when the BS2 signal impacts CEU2 and is constructively influenced by the STAR-RIS arrangement. Suggesting that ASTAR’s benefit fluctuates substantially depending on the operation of STAR-RIS (boosting or canceling the signal), the channel conditions, and the CEU location. Moreover, at CEU2, the Genie-Aided implementations of ASTAR and NP-STAR outperform their DL equivalents by only a small margin. This comparison demonstrates that ASTAR’s performance advantage over NP-STAR varies greatly by P_2_ and CEU location, with a significant benefit at CEU1 and approximately equal performance at CEU2 once appropriate STAR-RIS elements are deployed.

In addition, Table 3 illustrates the spectral energy efficiency (SEE) performance measured in KBits/J, at CEU1 with P_2 _= − 10 dBw, as a function of the STAR-RIS element count. The comparison of NP-STAR and ASTAR reveals distinct performance patterns when using DL Reflection Beamforming techniques. In the NP-STAR configuration, SEE consistently increases as the number of STAR-RIS elements rises from 8 to 64 that is because the achievable data rate is enhanced, and hence, this improves the SEE despite the increased consumed power with increasing elements. The maximum SEE recorded is approximately 5405 KBits/J at 64 elements.

**Table 3:** Comparison of SEE Performance in MBits/J for different STAR-RIS elements using NP-STAR vs ASTAR (P_2_ = − 10 dBw at CEU1).

Number of STAR-RIS elements	NP-STAR SEE (KBits/J)	ASTAR SEE (KBits/J)
8	~ 5320	~ 5270
16	~ 5360	~ 5310
32	~ 5390	~ 5350
64	~ 5405	~ 5305

**Fig. 9:**
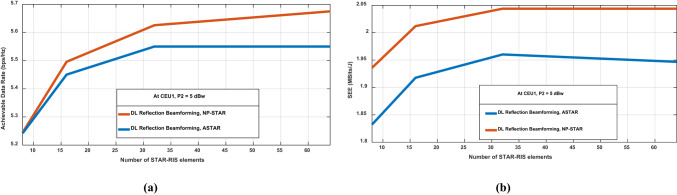
Comparison between the proposed ASTAR and NP-STAR at CEU1 with P_2_ = 5 dBw versus STAR-RIS number of elements for (**a**) achievable data rate. (**b**) SEE.

In contrast, the ASTAR implementation exhibits a non-monotonic relationship. SEE initially rises with more STAR-RIS elements, peaking at around 5350 KBits/J with 32 elements, then declines as the count approaches 64. Table 3 suggests that ASTAR has an optimal operating point that balances energy efficiency with system complexity. It is clear from Eq. (18) that the consumed power in the ASTAR is more sensitive to $M$, especially when adding more elements to the ASTAR, which needs more power amplifiers and phase shifters. As a result, the SEE for ASTAR illustrates that as M increases with ${P}_{PS}=10 mw$, the consumed power by the phase shifter and power amplifiers becomes significant, leading to a larger consumed power ${P}_{C}$, as explained in Eq. (18), which subsequently decreases the SEE after a threshold of M, despite increasing the achievable data rate. For NP-STAR, as the number of elements increases, power consumption also rises according to Eq. (16). However, the increase in M also enhances the achievable data rate, with a limit that enables it to mitigate the impact of increased power consumption. The table findings indicate that NP-STAR configurations benefit from maximizing the number of STAR-RIS elements within the tested range, while ASTAR implementations require careful optimization to find the ideal element count. The differing behaviors emphasize the need for system-specific design considerations in deploying STAR-RIS technology in practical wireless communication scenarios. This indicates that, while ASTAR may present other benefits, NP-STAR significantly outperforms ASTAR in spectral energy efficiency, exceeding it by more than an order of magnitude at the tested power level and user equipment location. In addition, the performance metrics of ASTAR and NP-STAR across different numbers of STAR-RIS elements at P_2_ = 5 dBw are compared and illustrated in Fig. 9, providing insights into system behavior for transmit powers of BS2 controlling and studying its effect on CEU1. In Fig. 9a, depicting achievable data rates at CEU1, both configurations show notable performance gains as the STAR-RIS number of elements increases from 8 to 64. The NP-STAR implementation in Fig. 9a consistently outperforms ASTAR, achieving peak data rates of approximately 5.69 bps/Hz at 64 elements, compared to ASTAR’s 5.57 bps/Hz.

NP-STAR continues to improve gradually beyond 32 elements, while ASTAR performance levels off after around 32 elements. Meanwhile, Fig. 9b presents the SEE in MBits/J at CEU1, revealing consequence efficiency trends. The NP-STAR implementation excels in energy efficiency, reaching about 2.05 MBits/J at 32 elements before stabilizing, while ASTAR peaks at approximately 1.96 MBits/J at the same element count, then slightly declines with further increases in element number. The Fig. 9a,b findings underscore a key design verification that in the high-interference scenario, NP-STAR offers higher data rates as justified in Fig. 4b, in addition to achieving better energy efficiency. Deployment decisions should weigh the priorities of maximum throughput against energy consumption, which will be increased by increasing the STAR-RIS number of elements. Moreover, the diminishing returns beyond 30-35 elements for both configurations suggest an optimal balance for cost-effective system design, harmonizing performance improvements with implementation complexity.

Furthermore, the SR incorporates a sigmoid function to evaluate user satisfaction, as outlined in^41^. The rate satisfaction metric is defined by the following equation:

20 $${Sat}_{Rate}=\frac{1}{1+{e}^{-\delta (R/{R}_{threshold})}}.$$

${R}_{threshold}$ denotes the minimum required rate at the receiver, determined by factors such as the allocated resources, interference, transmission power, noise, and other variables. Meanwhile, $R$ represents the expected achievable data rate, and $\delta$ serves as a decision constant, influencing the steepness of the satisfactory curve. Lastly, ${Sat}_{Rate}$ is normalized on a scale from 0 to 1^42^.

**Fig. 10:**
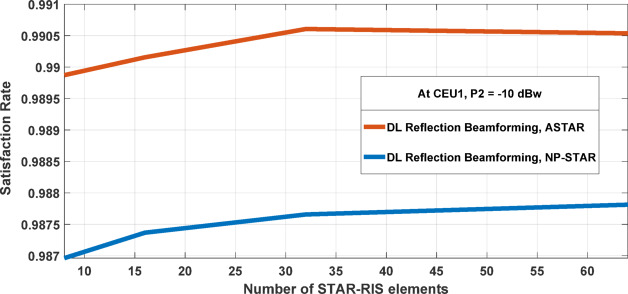
SR when expanding the number of elements in STAR-RIS from 8 to 64 UPA elements.

Figure 10 illustrates the Satisfaction Rate (SR) performance for ASTAR and NP-STAR configurations as the number of STAR-RIS elements increases from 8 to 64 UPA elements at CEU1, with a transmission power of P2 = − 10 dBw. As indicated in Eq. (20), the SR improves with more STAR-RIS elements due to enhanced data rates for users. User satisfaction is tied to achieving a data rate above the minimum threshold of 100 Mbps, and theory suggests that greater STAR-RIS deployment increases the likelihood of meeting or exceeding this benchmark. The graph shows that the ASTAR outperforms the NP-STAR configuration. ASTAR’s SR quickly nears 0.9905 with only a slight increase in STAR-RIS elements, rising from about 0.989 at 10 elements and stabilizing at approximately 0.9905 between 25 and 30 elements, and maintaining this high level up to 64 elements. At 64 elements, the SR for ASTAR approaches 0.962, demonstrating significant improvements from the M = 16 and M = 32 configurations. This highlights how increasing RIS elements enhances system performance and user satisfaction. Conversely, NP-STAR shows a gradual SR improvement, starting at around 0.987 with 8 elements and slowly rising to about 0.9885 as the element reaches 64. This conservative growth suggests that NP-STAR is less efficient in utilizing additional STAR-RIS elements compared to ASTAR. The marginal gains in both configurations beyond 30–35 elements indicate a potential optimal threshold for STAR-RIS design.

While increasing elements can enhance performance, returns diminish after a certain point. Researchers and engineers should weigh system complexity, implementation costs, and performance benefits when deciding on the optimal number of STAR-RIS elements for practical wireless communication systems.

**Fig. 11:**
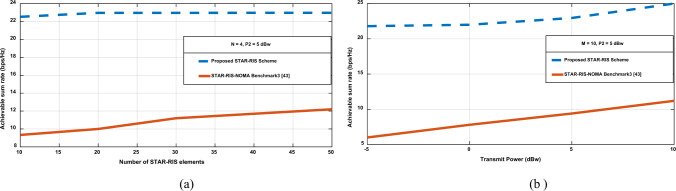
Achievable data rate (bps/Hz) comparison when changing (**a**) The number of elements in STAR-RIS. (**b**) The transmit power in dBw.

Figure 11 presents a comparative performance analysis between the proposed STAR-RIS-aided OFDM scheme and the STAR-RIS-NOMA benchmark from^43^. As shown in Fig. 11a, the proposed scheme achieves a significantly higher data rate of approximately 23 bps/Hz compared to the 12.5 bps/Hz achieved by the STAR-RIS- NOMA system when utilizing 50 STAR-RIS elements, achieving an impressive 76% improvement. This enhancement stems from the innovative approach of independent user optimization and advanced interference management strategies tailored for each user. Specifically, the reflection-side user benefits from constructive interference, combining the STAR-RIS reflected signal with the LOS path, while the transmission-side user exploits destructive interference to mitigate unwanted signals from the non-serving BS. The consistent performance gap observed across the entire range of STAR-RIS elements (10–50) underscores the robustness of the proposed design. Similarly, Fig. 11b demonstrates that this performance advantage persists across various transmit power levels, with the gap widening at higher power configurations. These findings validate the effectiveness of integrating orthogonal resource allocation with customized interference management in multi-BS scenarios, outperforming conventional NOMA clustering methods in STAR-RIS-enabled networks. Notably, while the proposed scheme optimizes the system sum rate, the resource allocation between users is asymmetric, which could be particularly beneficial in accommodating heterogeneous quality-of-service requirements in next-generation wireless networks.

**Fig. 12:**
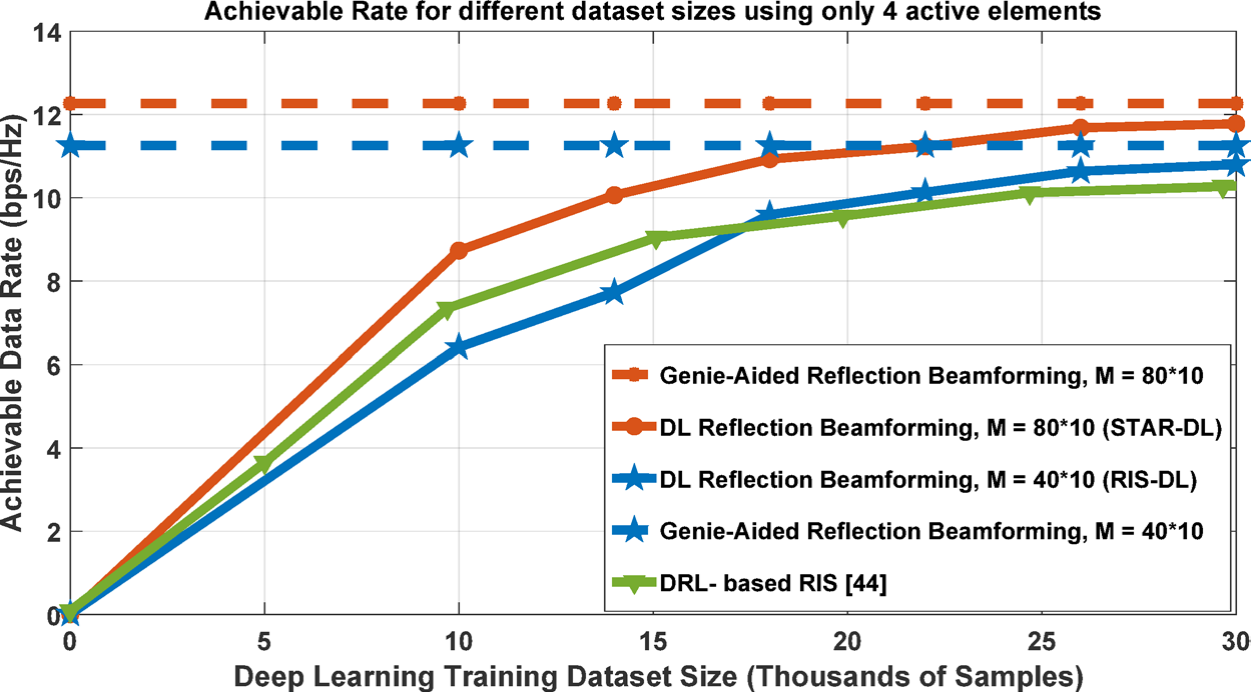
Achievable data rate analysis of STAR-RIS and conventional RIS systems utilizing DL-based beamforming optimization versus the RIS using DRL-based beamforming optimization at 3.5 GHz.

However, our proposed scheme necessitates more advanced resource allocation algorithms compared to NOMA-based systems due to its reliance on orthogonal frequency allocation and independent user optimization processes. The heightened computational complexity arises from the need to jointly optimize power allocation, frequency resource assignment, and STAR-RIS phase shifts across multiple users and base stations. In contrast, NOMA systems focus primarily on power domain multiplexing with simpler clustering mechanisms. Despite this increased complexity, the investment is justified by the substantial performance gains and the removal of successive interference cancellation requirements at user terminals, which simplifies receiver design and enhances reliability. Future research will aim to develop low-complexity algorithms and explore the trade-offs between complexity and performance in greater depth, ultimately providing practical implementation strategies for next-generation wireless networks. Additionally, Fig. 12 compares the DL-based STAR-RIS and DL-based RIS versus DRL-based RIS. The performance is evaluated against training dataset size for different methods.

These methods are the genie-aided reflection beamforming, DL-based reflection beamforming approaches (STAR-DL (M = 80 × 10) and RIS-DL (M = 40 × 10)), and a DRL-based RIS (M=40×10) baseline method^44^. Results show that STAR-RIS configurations consistently outperform conventional RIS. The STAR-DL approach achieves near-optimal performance (~ 12.2 bps/Hz) compared to DL-based RIS (~ 11.1 bps/Hz) after just 25,000 training samples, whereas the DRL-based RIS baseline reaches approximately 10.2 bps/Hz after 30,000 training dataset size. This demonstrates the clear advantage of supervised DL over reinforcement learning for this beamforming optimization problem. Notably, only 4 active elements are utilized across all configurations.

However, since the adopted DL is conventional and solely utilized for fair comparison, we have developed and implemented a novel spatial-temporal attention network (STAN) algorithm, which employs a spatial-temporal attention network to predict optimal beamforming configurations for STAR-RIS systems. Its primary innovation lies in transforming channel data into spatial grids, enabling the application of 2D convolutions for spatial attention, while fully connected layers effectively model channel attention mechanisms. The pseudocode shown in algorithm 2 outlines the core principles of the proposed STAN algorithm.

**Algorithm 2:**
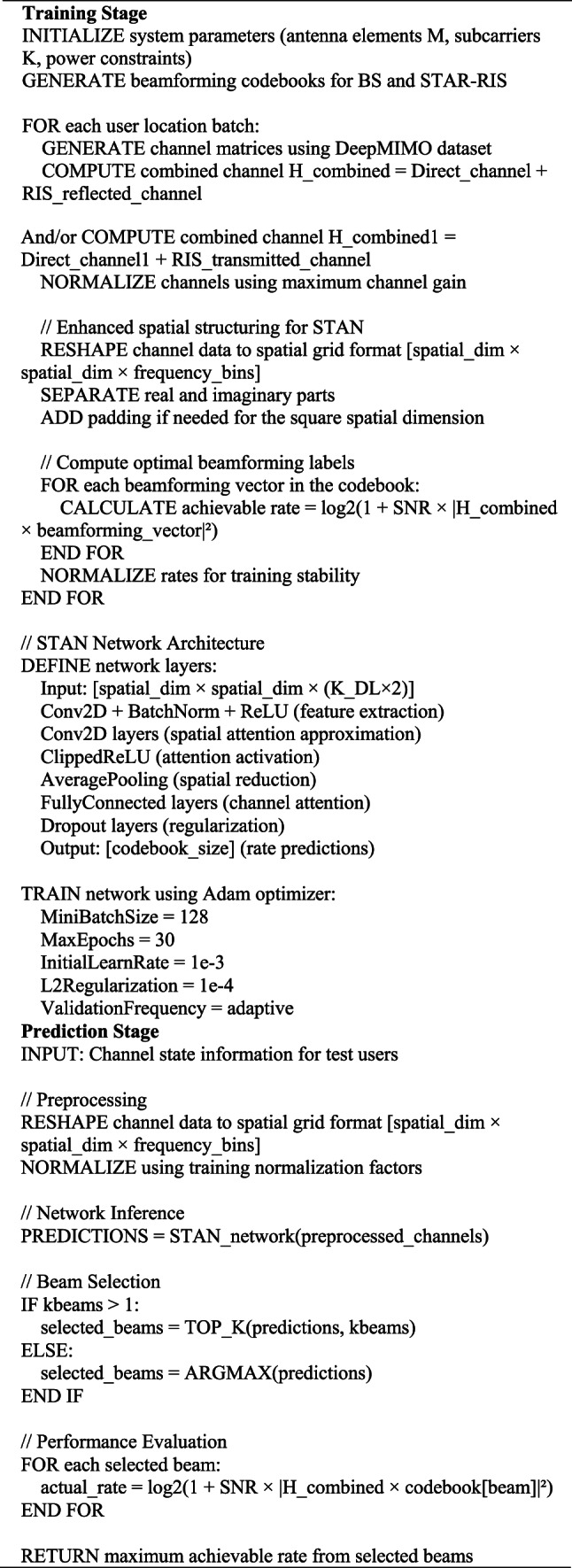
STAN (spatial-temporal attention network) pseudocode.

**Fig. 13:**
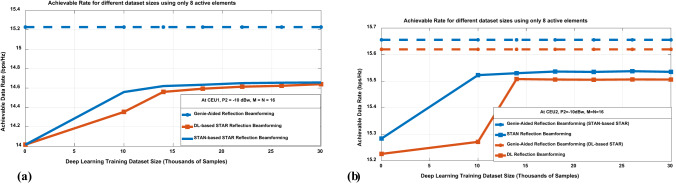
Performance comparison of achievable data rates using different beamforming approaches at (a) CEU1. (b) CEU2.

To evaluate the performance of our proposed STAN model against the conventional DL model, we analyze the achievable data rates at CEU1 and CEU2 with the size of the DL training dataset. Figure 13 highlights the performance superiority of the proposed STAN approach over the conventional DL method under various experimental conditions. In Fig. 13a for the CEU1 configuration, the STAN-based STAR outperforms the conventional DL approach, achieving near-optimal performance that closely aligns with the genie-aided benchmark that is the same for both techniques at CEU1. Notably, the performance gap between STAN and conventional DL methods increases as the training dataset size grows, showcasing the superior scalability of the STAN architecture.

Similarly, Fig. 13b for the CEU2 configuration reveals comparable trends where the STAN method demonstrates exceptional performance, consistently approaching the upper bound while maintaining a significant advantage over the conventional DL method across all dataset sizes.

Despite their theoretical benefits, both NP-STAR and ASTAR face significant real-world deployment hurdles. They are highly sensitive to the quality of CSI, necessitating frequent updates and precise estimations which expose performance vulnerabilities when CSI acquisition falls short of operational needs or estimation errors surpass acceptable limits. NP-STAR’s designs with high passive elements create a significant training bottleneck, as the extensive channel estimation demands may surpass the system’s coherence time. In addition, hardware implementation constraints pose considerable obstacles, especially for ASTAR systems that require dedicated power infrastructure and intricate switching mechanisms. The complexity of these components leads to increased costs, potential reliability issues, and timing constraints, which may hinder adaptation in rapidly changing environments. These factors present economic and operational barriers to widespread deployment. System scalability becomes challenging as computational complexity rises with larger array sizes and higher user densities. Current optimization algorithms may struggle to deliver real-time performance in large-scale deployments, while the lack of inter-cell interference coordination mechanisms complicates multi-cell environments, potentially affecting neighboring cells. Environmental adaptability and fault tolerance are critical weaknesses, as both systems lack robust mechanisms for graceful degradation during partial failures or significant operational shifts. The limited adaptation speed may be inadequate for high-mobility scenarios or dynamic interference conditions, risking performance collapse when conditions differ from training assumptions.

## Conclusion

This study provides a comprehensive analysis of the performance characteristics of STAR-RIS technology across diverse implementation strategies and deployment scenarios. The findings emphasize the critical importance of selecting between active and nearly passive elements, a decision that must be adapted to the specific interference environment of the deployment site. Active elements (ASTAR/ARIS) demonstrate superior performance in terms of achievable data rates, spectral energy efficiency, and satisfaction rate under favorable channel conditions. However, their tendency to amplify interference can hinder performance in high-interference environments. The study also underscores the influence of training dataset size on system performance, showing that DL-based approaches can effectively close the gap with genie-aided upper bounds, especially in challenging channel conditions. This suggests that practical implementations can achieve near-optimal performance with sufficient training data. Additionally, an analysis of element scaling reveals rapid initial performance improvements that gradually diminish, indicating an economic sweet spot for deployment scale. Transmit power configuration emerged as another key factor in optimizing system performance. The results illustrate how power adjustments can effectively balance trade-offs among users in different locations. The spectral energy efficiency (SEE) degradation of active implementations regards the nearly passive one validates their higher energy consumption. Finally, these insights deepen our understanding of STAR-RIS technology and offer actionable guidance for system designers. Future research should focus on developing adaptive control mechanisms that can dynamically transition between active and nearly passive modes based on real-time interference conditions. A particularly promising approach involves Reinforcement Learning-Based Mode Switching. Techniques such as Deep Q-Networks (DQN) or Multi-Armed Bandit algorithms could be employed to derive optimal switching policies by framing mode selection as a sequential decision-making problem. The reinforcement learning agent would assess variables such as channel conditions, interference levels, and power constraints, then strategically choose between active and passive modes to optimize a reward function that balances spectral efficiency and energy consumption. Such advancements could unlock even greater performance potential across a wide range of deployment scenarios.

## Data Availability

The datasets used and/or analyzed during the current study are available from the corresponding author on reasonable request.
